# Effect of insecticide-treated bed nets on visceral leishmaniasis incidence in Bangladesh. A retrospective cohort analysis

**DOI:** 10.1371/journal.pntd.0007724

**Published:** 2019-09-16

**Authors:** Rajib Chowdhury, Vashkar Chowdhury, Shyla Faria, Sakila Akter, Aditya Prasad Dash, Sujit Kumar Bhattacharya, Narayan Prosad Maheswary, Caryn Bern, Shireen Akhter, Jorge Alvar, Axel Kroeger, Marleen Boelaert, Qamar Banu

**Affiliations:** 1 International Center for Diarrhoea Disease Research, Bangladesh (icddr,b), Mohakhali, Bangladesh; 2 National Institute of Preventive and Social Medicine, Mohakhali, Bangladesh; 3 Department of Statistics, Dhaka College, Bangladesh; 4 Directorate General of Health Services (DGHS), Mohakhali, Bangladesh; 5 Central University of Tamil Nadu, India; 6 Kothari Medical Centre, West Bengal, India; 7 UCSF School of Medicine, San Francisco California, United States of America; 8 Drugs for Neglected Diseases initiative (DNDi), Geneva, Switzerland; 9 Special Programme for Research and Training in Tropical Diseases, World Health Organization, Geneva, Switzerland; 10 University of Freiburg, Centre for Medicine and Society/Anthropology, Freiburg, Germany; 11 Institute of Tropical Medicine, Belgium; 12 Asian University for Women, Chittatong, Bangladesh; National Institutes of Health, UNITED STATES

## Abstract

**Background:**

Visceral leishmaniasis (VL) is a parasitic disease, transmitted by the sand fly species *Phlebotomus argentipes* in the Indian sub-continent. Effective vector control is highly desirable to reduce vector density and human and vector contact in the endemic communities with the aim to curtail disease transmission. We evaluated the effect of long lasting insecticide treated bed nets (LLIN) and bed nets impregnated with slow-release insecticide tablet K-O TAB 1-2-3 (jointly insecticide-treated nets or ITN) on VL incidence in a highly endemic sub-district (upazila) in Bangladesh.

**Methods:**

Several distributions of LLIN or K-O TAB 1-2-3 for self-impregnation of bed nets at home took place in Fulbaria upazila, Mymensigh district from 2004 to 2008 under three research projects, respectively funded by CDC, Atlanta, USA (2004) and WHO-TDR, Geneva, Switzerland (2006 & 2008). We included all households (n = 8142) in the 20 villages that had benefited in the past from one of these interventions (1295 donated LLIN and 11,918 local bed nets impregnated with K-O TAB 1-2-3) in the “exposed cohort”. We recruited a “non-exposed cohort” in villages with contemporaneously similar incidence rates who had not received such vector control interventions (7729 HHs from nine villages). In both cohorts, we visited all families house to house and ascertained any VL cases for the 3 year period before and after the intervention. We evaluated the incidence rate (IR) of VL in both cohorts as primary endpoint, applying the difference-in-differences method.

**Results:**

The study identified 1011 VL cases (IR 140.47/10,000 per year [py]) before the intervention, of which 534 and 477 cases in the intervention and control areas respectively. The IR was 144.13/10,000 py (534/37050) and 136.59/10,000 py (477/34923) in the intervention and control areas respectively, with no significant difference (*p* = 0.3901) before the intervention. After the intervention, a total of 555 cases (IR 77.11/10,000 py) were identified of which 178 (IR 48.04/10,000 py) in the intervention and 377 (107.95/10,000 py) in the control area. The intervention area had a significant lower IR than the control area during follow up, rate difference = –59.91, *p*<0.0001. The IR during follow up was significantly reduced by 96.09/10,000 py in the intervention area (*p*<0.0001) and 28.63/10,000 py in control area (*p*<0.0001) compared to baseline. There was a strong and significant overall effect of the ITN intervention, *δ =* –67.45, *p* <0.0001. Sex (OR = 1.36, *p*<0.0001) and age (OR = 0.99, *p*<0.0001) also had a significant effect on VL incidence. Male had a higher risk of VL than female and one year increase in age decreased the likelihood of VL by about 0.92%. Two third of the VL incidence occurred in the age range 2 to 30 years (median age of VL patients was 17 years).

**Conclusion:**

VL incidence rate was significantly lower in the ITN intervention cohort compared to control in Bangladesh. Some bias due to more intense screen-and-treat activities or other interventions in the intervention area cannot be ruled out. Nonetheless, given their feasibility and sustainability, ITNs should be considered for integrated vector control during the maintenance phase of the VL elimination programme.

## Author summary

Visceral leishmaniasis (VL) is a deadly parasitic disease, transmitted by the sand fly species *Phlebotomus argentipes* in the Indian sub-continent. Humans are the only proven reservoir of the parasite, *Leishmania donovani*. Effective vector control is highly desirable to reduce vector density and human and vector contact in the endemic communities to stop the disease transmission. We evaluated the effect of long lasting insecticide treated bed nets (LLIN) and bed nets impregnated with slow-release insecticide tablet K-O TAB 1-2-3 (jointly insecticide-treated nets or ITN) on VL incidence in a highly endemic sub-district (upazila) in Bangladesh. The nets were either donated or impregnated between 2004 to 2008 under three studies and defined as “exposed cohort” comparing their effect on VL incidence with “non-exposed cohort” (no donation of impregnated nets) for a 3 year period before and after the intervention. The study identified 1011 VL cases (IR 140.47/10,000 per year [py]) before the intervention, of which 534 and 477 cases in the intervention and control areas respectively. There was a strong and significant overall effect of the ITN intervention, *δ =* –67.45, *p* <0.0001. The VL incidence rate was significantly lower in the ITN intervention cohort compared to control in Bangladesh, though some bias cannot be totally ruled out.

## Introduction

Visceral leishmaniasis (VL)—also known as kala-azar (KA) in the Indian sub-continent—is a deadly parasitic disease transmitted by the female *Phlebotomus argentipes* sand fly. In the South-East Asia Region, humans are the only proven reservoir of the parasite, *Leishmania donovani*. Kala-azar has been present in the Bengal territory (presently West Bengal, India, and Bangladesh) since the early 1800s [1] and gradually spread along the course of the Ganges and the Brahmaputra rivers, the major transport routes of Bengal. In what is today Bangladesh, KA was first described in 1824 in Jessore district [2], where an epidemic killed an estimated 75,000 people between 1824 and 1827 [1]. The historical records describe the classical picture of KA, with prolonged irregular fever, progressive emaciation despite good appetite, enlargement of liver and spleen and black coloration of skin [3].

In the late 1950s and early 1960s, WHO launched a malaria eradication programme throughout the South Asian sub-continent based primarily on Indoor Residual Spraying (IRS) of Dichlorodiphenyltrichloroethane (DDT). During this programme, KA almost disappeared as a collateral benefit [4]. However, within a few years after the end of the malaria eradication efforts, KA returned to Bihar and Bengal on both sides of the India-Bangladesh borders [5].

In Bangladesh, sporadic KA cases were reported again from the late 1960s onwards [6]. Between 1968 and 1980, 59 KA patients were reported, mostly from 5 districts (Sirajgang, Pabna, Mymensingh, Rajshahi, and Tangail) [7]. The numbers of KA cases soared from 1980 onwards, and a major outbreak occurred in Pabna district [1]. Between 1994 and 2013, the National Programme of Disease Control, Directorate General Health Services (DGHS), Government of Bangladesh reported 109,266 KA cases and 329 deaths [8]. Fifty percent of those cases were reported from just five sub-districts (Upazila) of Mymensingh district [8].

In 2005, three countries (Bangladesh, India, and Nepal), supported by WHO, launched a regional initiative to eliminate KA as a public health problem from the region and signed a Memorandum of Understanding (MoU) to this effect. The initiative aimed to reduce the KA incidence to one case per 10,000 population in each endemic sub-district by 2015 [9]. This deadline was later extended to 2017 [10], and 2020 in the London Declaration on Neglected Tropical Diseases [11]. Despite an impressive decrease in the number of cases in each country, WHO could not yet validate the KA elimination status in any of them and advocates for more intense and sustained control efforts and disease surveillance.

The intervention strategies in the elimination programme are based on case detection and treatment and integrated vector management (IVM) [9]. In Bangladesh, however, no specific sand fly control operations were carried out by the programme between 1999 and early 2012 [8,12]. It took a long time to register the required insecticides for the indication of sand fly control. The first indoor residual spraying (IRS) activity was conducted using deltamethrin 5WP in April/May 2012 in eight highly endemic Upazilas (sub-districts) [13]. Till today none of the countries was able to fully implement the IVM strategy in the region, as they tend to implement IRS for sand fly control only, and this in an independent way of any other vector control operation. Although well-performed IRS can reduce vector density dramatically, it remains operationally challenging and expensive, and its acceptance by the community is not always optimal. Several authors have highlighted its limitations, in terms of insecticide resistance, quality of implementation, occupational hazard, cost, sand fly adaptation, etc. [14–17]. Therefore, there is a need for alternative tools which are operationally easy to implement and cost-effective in terms of per household protected. The question of whether there are alternatives to IRS will only become more relevant in the post-elimination era. We briefly summarize here the evidence on *P*. *argentipes* control methods from Bangladesh and the region so far. Between 2002 and 2009 several epidemiological and entomological studies were conducted in the highly endemic area of Fulbaria, one of the Upazilas of Mymensingh district, either to assess the KA disease burden and its risk factors [18], or to evaluate the effectiveness of insecticide-treated nets as an alternative for IRS for controlling the *P*. *argentipes* sand fly [19–21]. Consistent use of non-treated local bed nets in summer was associated with reduced risk for VL in an observational study [18]. This study also showed that use of bed nets is acceptable in the rural community of Bangladesh and found a high percentage of households owning at least one bed net [18], similar to evaluations in India and Nepal [22].

Inspired by the effectiveness of insecticide treated bed nets for malaria control, several intervention studies evaluated either donated long-lasting insecticide-impregnated bed nets (LLIN) or local bed nets impregnated with slow release insecticide tablet K-O TAB 1-2-3, on entomological endpoints [19–21]. For ease of understanding, we regroup both interventions as “Insecticide-Treated Nets” (ITN) in the remainder of the text. The two multi-country intervention studies found significant reductions in sand fly density, ranging from 60% to 80%. A less pronounced 25% reduction of sand fly density was found in a cluster randomized trial (CRT) conducted in India and Nepal comparing households covered by LLIN with households where no LLINs were used, which were allowed to continue to use their own commercially available non-treated nets [23]. However, the CRT study in India and Nepal did not find any effect of the LLIN distribution on *Leishmania donovani* infection nor KA incidence, notwithstanding a high coverage of all household members and effective use of the LLINs [24]. Authors suggested this negative finding might be related to exposure outside the peridomestic environment due to changing sand fly behaviour, which was partly confirmed later [25]. Long-standing insecticide pressure because of the repeated IRS campaigns in India and Nepal might have forced sand flies to adapt again to the outdoor environment.

It is worthwhile to study the same question in Bangladesh though, as the sociocultural and environmental parameters are somewhat different. In Bangladesh, in contrast to India, no IRS was in place for a very long time in the KA endemic areas, so there was no insecticide pressure on the peridomestic sand fly habitat. Therefore, we set out to investigate the impact of ITNs on KA incidence in Bangladesh through a retrospective cohort analysis, as staging another CRT would raise ethical questions and would not be feasible in the present context of very low incidence rates near elimination.

## Methods

### Ethics statement

The present study protocol was approved by the Ethical Review Committees of the Bangladesh Medical Research Council (BMRC) and the Special Program for Research and Training in Tropical Diseases/Regional Office for South-East Asia, World Health Organization (WHO/SEARO), India. Informed consent in the household survey was signed by the head of household before their voluntary participation in the study.

### Study design and population

This study is a retrospective cohort analysis set in Fulbaria sub-district, Bangladesh. Fulbaria is located 111 km from the capital city Dhaka, and 23 kilometers away from the district headquarters respectively. Fulbaria has 13 unions (lowest administrative unit) and 116 villages. The Upazila occupies an area of 398.70 km^2^ including 14.76 km^2^ forest area.

In the Fulbaria population, we retrospectively defined two distinct cohorts; the exposed cohort was the one who benefited from an ITN intervention in the recent past, and the unexposed were those who did not. The first, “**exposed cohort”** was therefore composed of all the communities who had benefited previously from a LLIN or K-O TAB 1-2-3 distribution in 1 of three distinct studies (18–21). The **non-exposed cohort** (control) (i.e., families that did not receive any donated long-lasting nets or whose local nets were not impregnated), was composed of villages of similar population size with a comparable KA incidence rate in the corresponding study period of each of the three published studies (18–21).

**Fig 1:**
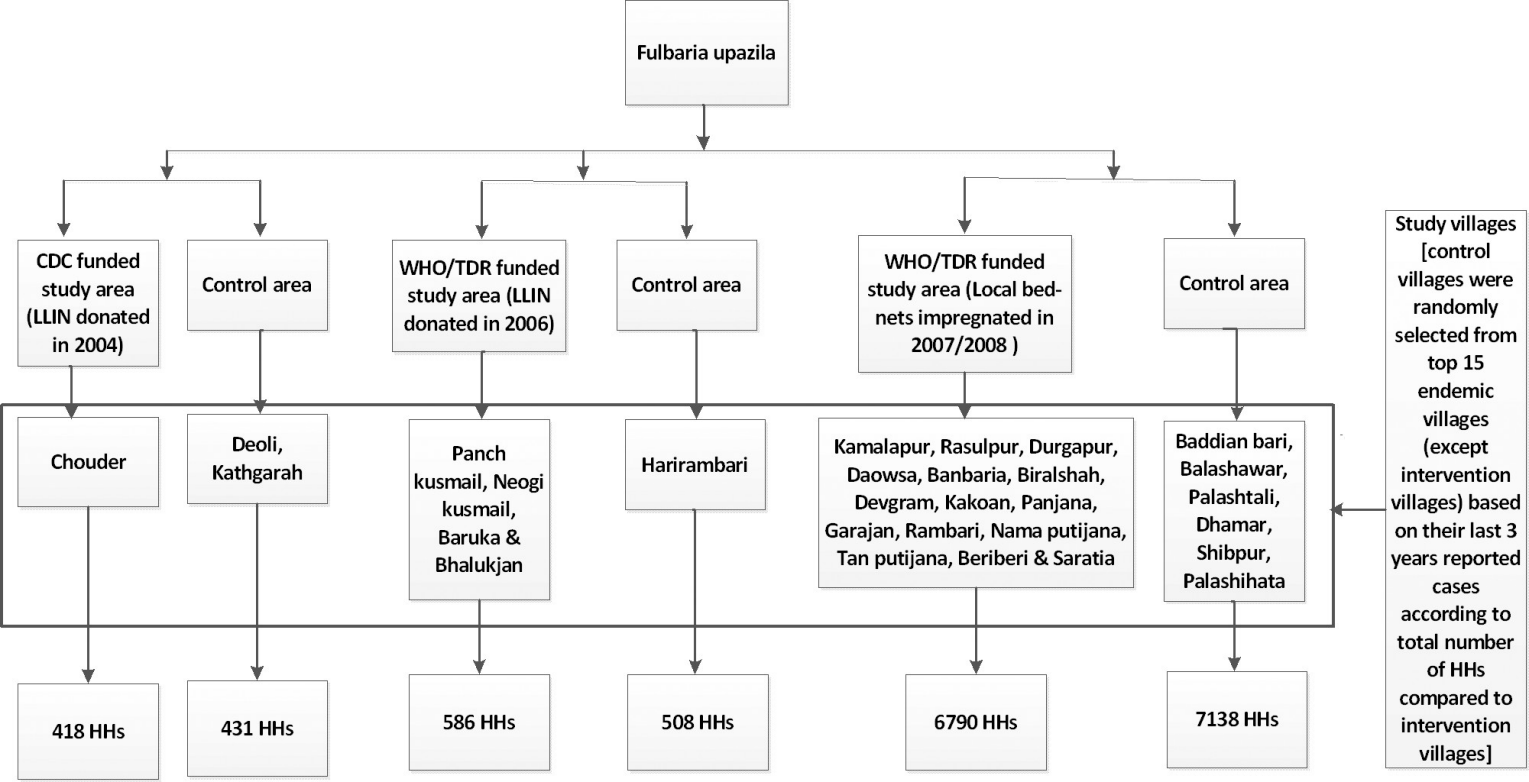
Study design [HH = household].

Based on data from the epidemiological records of the Ministry of Health (passive surveillance data), we then ranked all the KA endemic villages of Fulbaria (excluding those already included in the exposed cohort), according to the number of reported cases, for the corresponding time period when the respective ITN interventions were carried out (2004, 2006, 2008). We then randomly selected nine endemic villages from the 15 top-ranked villages (Baddiyan bari, Balashawr, Palashtali, Deoli, Dhamar, Shibpur, Kathgara, Harirumbari, and Palashihata) (Fig 1), and included these nine communities (n = 7729) in the control cohort.

### Exposure to past ITN interventions

We describe here the different ITN interventions that took place in the exposed cohort. A first epidemiological study was conducted in a total of 506 households from three paras (sub-villages) namely Nadirpar, Lakxmipur, and Bamonbaid of Chouder village (Fig 1) of Fulbaria union, Fulbaria Upazila, Mymensingh district between 2002 and 2004 [18]. The study was funded by CDC, Atlanta, USA and implemented by icddr,b. After completion of the study, each HH was donated one unit of LLIN (manufactured by Vestergaard Frandsen Private Limited).

Between 2006 and 2007, a cluster randomized trial was conducted with four arms (3-intervention [arm-1: IRS using deltamethrin 5 WP, arm-2: LLIN and arm-3: environmental management] and 1-control arm where LLIN were donated after completion of the study period) in Fulbaria Upazila (Fig 1), Mymensingh district [19, 20]. This study involved a total of 596 households. The study was funded by the Special Programme for Research and Training in Tropical Diseases (TDR), WHO, Geneva, Switzerland and conducted by the National Institute of Preventive and Social Medicine (NIPSOM), Bangladesh. LLIN (manufactured by Vestergaard Frandsen Private Limited) were donated in two arms.

Last, a community-based study was conducted with 6967 households in Putijana union (Fig 1) of Fulbaria Upazila, Mymensingh district between 2007 and 2008 [21]. In this study, all existing bed nets at HH level were impregnated with slow release insecticide tablet K-O TAB 1-2-3 (0.4g deltamethrin in a 1.6 g tablet and a chemical binder) manufactured by Bayer Crop Science, Isando, South Africa. The study was supported by the Special Programme for Research and Training in Tropical Diseases (TDR), WHO, Geneva, Switzerland and conducted by National Institute of Preventive and Social Medicine (NIPSOM), Bangladesh.

### Ascertainment of outcome data

We assessed the outcome “KA” independently from the original research projects, and in the same way for the intervention and control area, as follows. We exploited the full database for Fulbaria sub-district for the period of 2001 to 2011 to identify reported KA cases in the intervention and control areas and visited all affected communities in an exhaustive house to house survey. All households (HH) of both cohorts were visited between 2011 and 2012, and the head of the HH/responsible adult was interviewed in order to ascertain the number of reported KA cases in the period of three years before and after the intervention for the three distinct study sites described above, and in a matching time frame for the control cohort. The period of observation was seven years for each intervention, and the respective time windows were as follows: for the 2004 CDC funded study: 2001–2007; for the 2006 TDR study: 2003–2009; and for the 2008 TDR study: 2005–2011. Additionally, information about current bed net use and washing practices was also collected in the intervention area. Trained Research Assistants conducted the interview using a structured questionnaire.

### Data management and statistical analysis

A standard data entry interface was designed using Microsoft Office Access for entering study data. Data were checked and cleaned before analysis. Percentages were used to summarise the demographic and study variables. VL incidence rate was calculated for control and intervention areas (per 10,000 persons per year) for baseline and follow-up period separately. Z-test was used to compare the VL incidence rates between the intervention and control area, and p-values at the 0.05 significance level were used. Difference In Difference (DID) estimates (δ) were calculated to estimate the effect of the intervention at the community level. Binary logistic regression was used to calculate odds ratios for the effect of gender and age on VL incidence rate. STATA/MP 13.0 for Windows (StataCorp LP, College station, TX77845, USA) was used for data analysis.

## Results

### Study population characteristics

Of a total of 15,871 HHs (71,973 population), 8142 HHs (37,050 population) and 7729 HHs (34,923 population) were included in the study as exposed and control cohort. Table 1 shows that their baseline characteristics are very comparable, including for the frequency of KA at baseline in household level.

**Table 1:** Characteristics of study population in Fulbaria upazila, Mymensingh district, Bangladesh.

Variable	Category	Intervention area, n (%)	Control area, n (%)
Number of households		8142	7729
Population		37050	34930
Sex	Male	18930 (51.09)	17859 (51.13)
	Female	18120 (48.91)	17062 (48.85)
Age	0–20 years	18328 (49.47)	16985 (48.63)
	21–40 years	11000 (29.69)	10493 (30.04)
	41–60 years	5447 (14.70)	5375 (15.39)
	61+ years	2277 (6.15)	2067 (5.92)
Education	Illiterate	15818 (42.69)	15874 (45.45)
	Primary or below primary	18240 (49.23)	15857 (45.40)
	Secondary or Higher secondary	2502 (6.75)	2565 (7.34)
	Graduate or Higher	490 (1.32)	624 (1.79)
Profession	Housewife	9258 (24.99)	8940 (25.59)
	Student	8937 (24.12)	8869 (25.39)
	Labour	5480 (14.79)	4170 (11.93)
	Farmer	3449 (9.31)	4279 (12.25)
	Business	1192 (3.22)	1186 (3.40)
	Government/non government job	622 (1.68)	646 (1.85)
	Jobless or retire or others	2492 (6.73)	2089 (5.98)
	0–6 years children	5620 (15.17)	4735 (13.56)
Proportion of households affected by KA before intervention	Minimum 1 KA case in 3 previous years	446 (5.48)	413 (5.34)

### Information on bed nets and their use in the intervention cohort

In the household survey, we investigated the persistent use of bed nets in the intervention area. Of 8142 HHs that benefited at one point in the past from an ITN distribution in the intervention area, more than 92.2% HHs had at least one bed net in their house at the time of our household survey. Among those, 80.1% were ITNs, either self-impregnated with K-O TAB 1-2-3 or LLIN, the others were non-impregnated commercial nets (Table 2). About 33.9% HHs even had two nets, of which 88.9% were ITNs. However, 7.8% of all HHs did not have any bed net at the time of survey. More than 84.3% HHs (6864/8142) informed that they were always sleeping under a bed net. Sixty-five percent of all HHs reported that they felt impregnated nets were effective against mosquitoes along with other insects while about 32% HHs informed nets only effective against mosquitoes. About 82% HHs stated that there were less kala-azar (VL) cases in their community after the introduction of impregnated nets while about 17% respondents had no opinion. Only 12% of our respondents knew that kala-azar is transmitted by a sand fly bite, while the majority (74.4%) said it is transmitted by mosquitoes. Almost all HHs (98%) expressed a demand for ITNs, and the majority (72.7% respondents) asked for a free-of-cost distribution as a government donation (Table 2).

**Table 2:** Information on bed net and knowledge of disease transmitting agent of intervention areas of Fulbaria upazila, Mymensingh district, Bangladesh.

Statements	N, (%)
Number of families having bed nets	
*1 net*	3063 (37.6); of them 2502 impregnated
*2 nets*	2764 (33.9); of them 2454 impregnated
*3 nets*	1225 (15.0); of them 1137 impregnated
*4 nets*	343 (4.2); of them 321 impregnated
*More than 4 nets*	114 (1.4); of them 111 impregnated
*Not having net*	633 (7.8)
Reason for having un-impregnated net in families	
*During impregnation/distribution not at home*	422 (5.2)
*Buy new net due to torn*	1915 (23.5)
All family members sleep under net	
*Yes*	6867 (84.3)
*No*	1275 (15.7)
Reason for not sleeping under net	
*Insufficient net*	491 (38.5)
*Torn net*	789 (61.9)
Comments on net by household	
*Effective against mosquitoes and all insects*	5312 (65.2)
*Only effective against mosquitoes*	2583 (31.7)
*Not effective at all*	248 (3.1)
Using impregnated net	
≤*5 years*	6923 (85.0)*
*More than five years*	379 (4.7)
Did kala-azar reduce in your community because of net use	
*Yes*	6658 (81.8)
*No*	123 (1.5)
*Don’t know*	1361 (16.7)
Which insect transmits kala-azar	
*Sand fly*	962 (11.8)
*Mosquito*	6056 (74.4)
*Others*	1125 (13.8)**
Willingness to re-impregnate/new net	
*Yes*	7981 (98.0)
*No*	162 (2.0)
Willingness to pay for re-impregnation, how much can spend (in USD)	Up to USD 1 (24.4) Want free government donation– 5918 (72.7)

*In Putijana union, 5.3% HHs net become useless within two years after the impregnation due to torn and 10.3% HHs not have net

**Others = spider, dengue mosha (*Aedes* mosquito), dusito pani (polluted water), kharap batas (polluted air)

ITNs had been washed upto 5 times in 57.6%, 23.0% and 67.5% of HHs and 6–10 times in 29.6%, 54.1% and 32.4% in Putijana union; Chouder village; and Bhalukjan, Panch Kushmail, Neogi Kushmail, Baruka villages respectively (Table 3). Regarding washing practice of nets, 88.5%, 88.2% and 94.9% HHs in the Putijana union; Chouder village; and Bhalukjan, Panch Kushmail, Neogi Kushmail, Baruka villages reported that they washed their nets in the pond (Table 3) which is not recommended. In Putijana union, the majority of the respondents (98.8%) said that they dried their nets in direct sunlight (also not recommended), while this was 76.5% and 53.9% in Chouder village and Bhalukjan, Panch Kushmail, Neogi Kushmail, Baruka villages respectively.

**Table 3:** Net washing practice in the intervention rural communities in Fulbaria upazila, Mymensingh district, Bangladesh.

Statements	Intervention areas		
	Putijana Union	Chouder Village	Bhalukjan, Panch Kushmail, Neogi Kushmail, Baruka Villages
	Findings N, (%)		
How many time washed nets since received or impregnated			
*Up to 5 times*	4112 (65.8)	96 (23.0)	396 (67.5)
*6–10 times*	2114 (33.9)	226 (54.1)	190 (32.4)
*More than 10 times*	20 (0.3)	96 (23.0)	1 (0.1)
Net dried			
*In direct sun*	6168 (98.8)	319 (76.5)	316 (53.9)
*In shady place*	78 (1.2)	98 (23.5)	270 (46.1)
Where washed net			
*Tube well*	417 (6.7)	39 (9.4)	29 (5.0)
*Pond*	5530 (88.5)	351 (84.2)	556 (94.9)
*River/cannel*	299 (4.8)	27 (6.4	1 (0.1)
Net washing with			
*Detergent powder*	6087 (97.5)	394 (94.5)	586 (100)
*Soap*	159 (2.5)	23 (5.5)	00 (00)

### Effect of ITN

**Fig 2:**
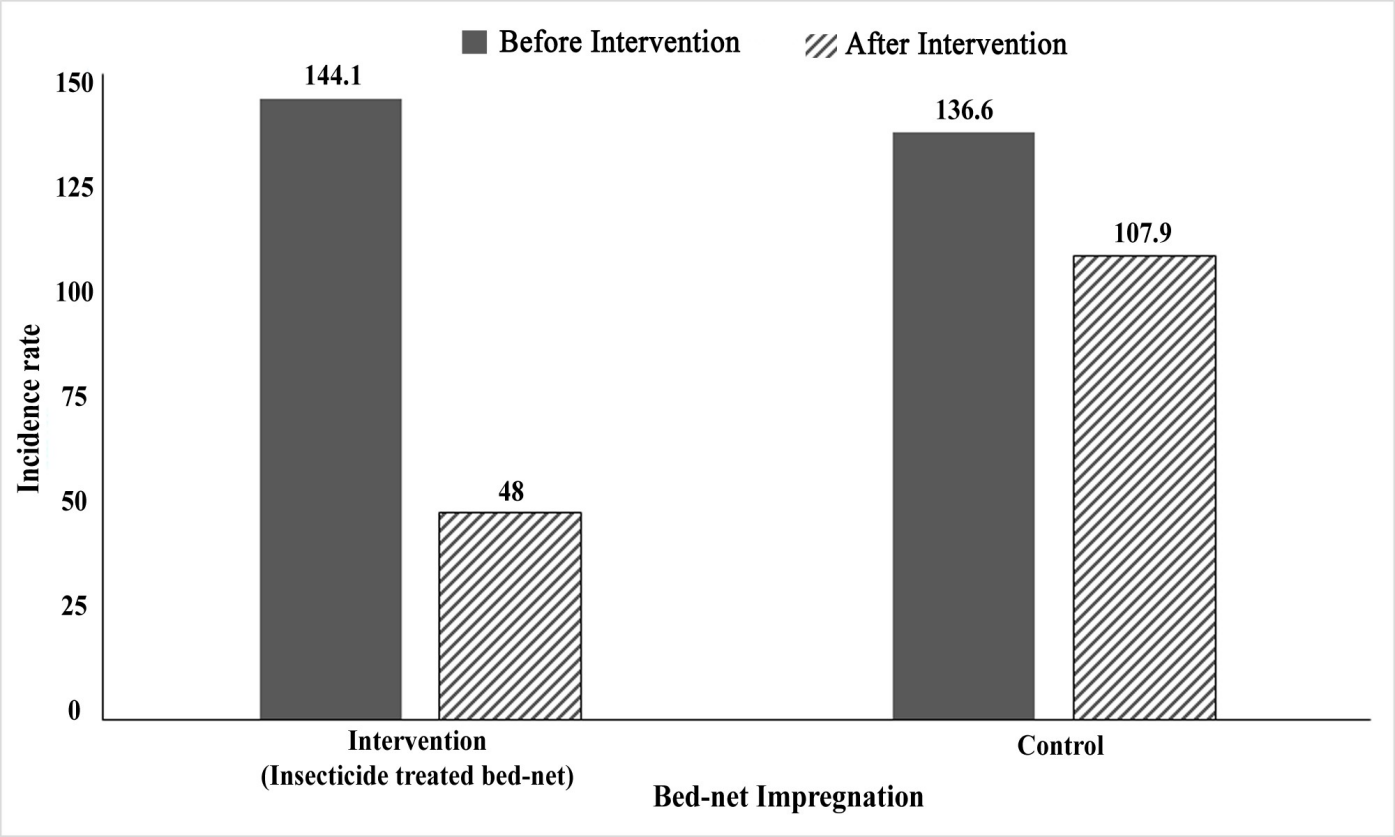
Visceral leishmaniasis incidence (KA cases per 10,000 population per year) in intervention and control areas before and after introduction of ITN in Fulbaria upazila, Mymensingh district, Bangladesh.

In the house-to-house survey, we recorded a total of 1011 VL cases (140.47/10,000/year) in the three years preceding the respective research projects of which 534 (144.13/10,000/year) and 477 cases (136.59 per 10,000/year) in the intervention and control areas respectively (Table 4; Fig 2). The difference in incidence rate (IR) was not statistically significant (*p* = 0.3901). In the three years after the research projects, we identified a total of 555 KA cases (incidence rate 77.11/10,000/year) of which 178 (48.04/10,000 per year) in the intervention area and 377 (107.95/10,000 per year) in control area (Table 4; Fig 2). The area that benefited from ITN had a significantly lower incidence rate than the control area in the 3-years follow up period, the rate difference was –59.91, *p*<0.0001. The VL incidence rate during follow-up was significantly reduced both in the intervention and control areas, by 96.09/10,000/year in intervention area (*p*<0.0001) and 28.63/10,000/year population in control area (*p*<0.0001) compared to baseline. The effect of the intervention was strongly significant, *δ =* –67.45, *p* <0.0001. The estimated reduction of VL incidence rate by the intervention was 46.80% (*p*<0.0001).

**Table 4:** Visceral leishmaniasis incidence rate before and after introduction of insecticide treated bed nets in Fulbaria upazila, Mymensingh district, Bangladesh compared to a contemporaneous control area without ITN distribution.

	Number of cases (Incidence Rate per 10,000 per year)		Rate change [After-Before]† (p value)	% reduction compared with control (p value)†
	Before intervention¶	After intervention¶		
Intervention n = 37050	534 (144.13)	178 (48.04)	-96.09 (<0.0001)	-46.80 (<0.0001)
Control n = 34923	477 (136.59)	377 (107.95)	-28.63 (0.0006)	
Total N = 71973	1011 (140.47)	555 (77.11)	-67.44 (<0.0001)	
Rate Difference (I-C)	7.54 (0.3901)	-59.91 (<0.0001)	-67.45 (<0.0001)	

*ITN = insecticide treated net, either a long lasting insecticide impregnated bed net or a KO TAB 1-2-3 self-impregnated net. Distributions were done in 2004, 2006 and 2008 respectively.

† Effect of intervention is calculated using difference-in-difference (DID) estimate, δ = (B–A)–(D–C), where A and B are the baseline value and post-intervention value for VL-affected HH per 1,000 HH/VL incidence per 10,000 persons in the intervention area respectively; C and D are the baseline value and post-intervention value for VL affected HH per 1,000 HH/VL incidence per 10,000 persons in the control area respectively. The effect of intervention is negative or positive if δ is negative or positive. Then the percentage reduction by intervention is calculated as [δ/A] × 100.

‡p values were calculated by Z statistic for pre- or post-rate differences between intervention and control areas.

Moreover, sex (OR = 1.36, *p*<0.0001) and age (OR = 0.99, *p*<0.0001) also had a significant effect on VL incidence. Male were more affected by VL than females. A one year increase in age decreased the likelihood of VL by about 0.92%. Seventy five percent of the VL incidence occurred in the age range of 2 to 30 years (median age of VL patients was 17 years).

## Discussion

The main finding of our analysis is that the introduction of ITN in rural highly endemic communities in Bangladesh was associated with a significantly greater reduction of the VL incidence, compared to unexposed communities that also experienced a reduction over time but of lesser size.

The present study confirmed that the use of bed nets is a common practice in the rural community of Bangladesh as observed by others [18]. We found that many households in the intervention cohort were still using the nets which had been distributed during the previous studies. A certain proportion of HHs (about 8%) were not having bed nets, and those were most likely the poorest families, are mostly related to poverty as it is well established that VL affects the poorest communities in the Indian sub-continent (ISC) [26–28]. It has been observed that high coverage of bed net use has community effect on vector sand fly in India and Nepal [23], similar impact found for malaria vector in Tanzania [29], so unavailability of bed net in the small number of HHs might not have negative impact. However, the study findings suggest that the washing practices of the ITNs require some change to preserve their effectiveness. Impregnated nets should not dry in the direct sun light as no UV protection is in place in the net. Large number of people dried their nets in the direct sun in the intervention areas which may have reduced their efficacy. It is hard to explain why HHs dried their nets in the direct sun though they were informed to dry nets in the shady place. The possible reason could be HHs want to make sure bed net get dried before sunset in the same day of wash as they may not have extra net. Moreover, it is also recommended that impregnated net should not be washed in ponds or rivers as deltamethrin (synthetic pyrethroid) is poisonous for aquatic animals especially for fishes [30]. Unfortunately very few people washed their nets using tube well water. It is well established that VL endemic communities are poorest of the poor, due to this reason many of the study families may not have own tube well which forces them to wash their bed nets in the pond as it is convenient.

The strength of our design is that we were able to control for a declining temporal trend by comparing the effect in the intervention area with that of a contemporaneous control area. We also acknowledge two important limitations of our study design. As it is non-experimental in nature, there could be other factors that explain the trend in IRs in the cohorts, such as e.g. a more intense screen-and-treat as the baseline IR were of the highest in the region, and communities might have been targeted preferentially by the programme. We believe the influence of such factor to be minor, as prior to 2009 the elimination programme in Bangladesh was not yet in full swing [12]. At the time, except for some training, little governmental control activities took place. VL patients were in theory entitled to receive all medication free of cost in the government health facilities, but in practice there were severe drug shortages of Sodium Stibogluconate [18] until the introduction of Miltefosine as first-line treatment option in 2009 [8]. It later appeared that one of the batches of Miltefosine supplied by the national programme was a fake drug with no active substance [31], so we may consider that the effect of case management was minimal during that period. Similarly, no sand fly control activities were conducted by the government up to early 2012 [8, 12] since banned of DDT in 1997 [32], as the registration process of deltamethrin for sand fly control took a long time [13]. However, in 2013 the national programme distributed two pieces of LLIN to each patient who had completed VL treatment between 2009 and 2011 [8]. Secondly, our comparison is a one-to-one comparison of one cohort compared to another, and given the erratic behaviour of VL in small areas, the lack of replicates limits the robustness of our findings. Randomization of a sufficient number of study units to either intervention or control cohorts would undoubtedly lead to less biased results, but in the given context of very low case incidence, the organization of such trial is deemed not feasible.

Unfortunately, very few studies evaluated the impact of ITN on VL incidence in the ISC. The only study evaluating the impact of local nets impregnated with slow release insecticide on VL in Bangladesh found a 66.5% incidence reduction after one year of use in a comparison of one intervention to one control area [33]. Our study showed a significant reduction of VL incidence after three year of use. In Sudan, another observational study found a 59% reduction of VL after using impregnated bed net [34] which is in line with our findings as well. However, our findings contrast with those of pair-matched cluster randomized trial of LLIN distribution in India and Nepal where no VL incidence reduction was found [24]. However, the same study showed a significant reduction on malaria incidence, and the LLIN reduced about 25% *P*. *argentipes* sand fly density at household level [23]. The difference between Bangladesh and India/Nepal could be that long-term DDT spraying in India and synthetic pyrethroid spraying in Nepal induced some adaptation of sand fly behaviour towards more outdoor resting or feeding behaviour which is partially supported by a study from India [25].

To eliminate or control a vector-borne disease it is highly important to reduce human-vector contact and vector density. Till today except for IRS no other interventions are included in the vector control strategy of the VL elimination initiative. In the MoU, it was noted that IVM should be adopted as regional strategy for vector control, and this requires more than one tool [9]. Operationally IRS is a more challenging and also more expensive method than ITN distribution. Studies in Bangladesh, India and Nepal identified that the impact of IRS is sub-optimal when it was carried out by the national programme [13, 16]. Furthermore, VL cases are sharply reducing in the countries so that it will not be sustainable to continue blanket IRS operations in all endemic sub-districts in the country. Health authorities in the region may no longer allocate enough funding for IRS because they have many other health priorities to respond to. It is worth to mention that Bangladesh and Nepal so far did not receive any external funding to control the VL vector in contrast to India (personal observation, RC).

In this regard, the present study provides observational evidence of the effect of ITNs in the absence of other governmental control interventions. Given the affordability of ITNs [15], their ease of implementation and their acceptability, they should be given consideration for inclusion in integrated vector management, definitely in the era of post-VL-elimination [35,36].
